# Effect of Interleukin and Hepcidin in Anemia of Chronic Diseases

**DOI:** 10.1155/2020/3041738

**Published:** 2020-02-07

**Authors:** Maha F. Yacoub, Hala Fouad Ferwiz, Fadwa Said

**Affiliations:** ^1^Internal Medicine and Clinical Hematology, Faculty of Medicine, Cairo University, Giza, Egypt; ^2^El-Sahel Teaching Hospital, General Organization of Teaching Hospital, Cairo, Egypt; ^3^Clinical Pathology Department, Faculty of Medicine, Cairo University, Giza, Egypt

## Abstract

**Background:**

Anemia of chronic disease (ACD) also termed as the anemia of inflammation has been found to be associated with inflammations, chronic infections, and cancers, particularly in old age. Recent studies revealed that interleukin-6 (IL-6), a proinflammatory cytokine, and hepcidin, an antimicrobial hepatic peptide, play a key role in ACD pathogenesis. *Patients and Methods.* The study included 40 subjects with chronic diseases and 40 normal subjects of the same age group. Red cell indices, levels of IL-6 and hepcidin, and iron profile were measured in all participants using Bayer ADVIA 120, VITROS 5600, Integrated System/2008, and ELISA assay, respectively.

**Results:**

The level of hemoglobin was considerably less in patients of chronic diseases referred to as “cases” than the normal subjects or “controls” (8.7 ± 1.5 vs. 13.2 ± 0.9). Red blood corpuscle (RBC) count, hematocrit (HCT) level, serum iron, mean corpuscular hemoglobin concentration (MCHC), and serum total iron-binding capacity (TIBC) were found to be significantly lower in the cases as compared to controls (*p* < 0.001). Serum IL-6 and hepcidin levels were substantially higher in the cases than in the controls (*p* < 0.001). Serum IL-6 and hepcidin levels were substantially higher in the cases than in the controls (*p* < 0.001). Serum IL-6 and hepcidin levels were substantially higher in the cases than in the controls (

**Conclusion:**

This study detected a significant increase in serum IL-6 and hepcidin levels in patients with ACD than the controls. These findings offer an insight into the role played by both cytokine and peptide in the pathogenesis of ACD and thus provide a rationale for future use of novel drugs inhibiting their effects on iron metabolism.

## 1. Introduction

Anemia, which poses a serious health outcome, is a common disorder in elderly people [1]. Approximately 10% of adults over the age of 65 years and 20% over the age of 85 years tend to develop anemia [1]. Among the elderly patients with anemia, around 20% are considered to have anemia of chronic disease (ACD) or anemia of inflammation [2]. ACD is commonly observed in patients with chronic kidney disease, acute or chronic infection, malignancies, and inflammatory disorders such as rheumatoid arthritis [2–4]. ACD is the most prevalent form of anemia in patients staying in hospitals [5, 6]. Iron deficiency anemia is the most common anemia followed by ACD [7, 8]. The hallmark of ACD include moderate shortening of red blood corpuscle (RBC) lifespan, microcytic or normocytic iron deficiency anemia, decreased release of iron from cellular stores, low serum and preserved marrow iron, and inability of bone marrow to enhance erythropoiesis to cope with anemia [3, 9]. Typically, ACD patients develop normochromic and normocytic anemia; however, hypochromic and microcytic anemia has been found in 30–50% of the patients [9]. In ACD patients, mild anemia (about 2 g/dL reduction in hemoglobin) along with lowered mean cell volume (about 6 fL) and normal or marginally higher or lower reticulocyte count had been detected [9].

There are several complicated factors responsible for the development of ACD. Among them, the disturbance in iron homeostasis and iron distribution is the most predominant cause. Enhanced uptake as well as storage of iron occurs within the cells of the reticuloendothelial system, and less iron is released from the mononuclear phagocytic system [8, 10]. In addition, erythrophagocytosis plays a significant role in iron acquirement by macrophages [8]. Therefore, the level of circulating iron diminishes, which in turn reduces serum iron and lowers intestinal absorption of iron [8, 10]. Consequently, the erythroid progenitor cells receive less iron to produce erythrocytes to respond to anemia, and erythrophagocytosis lowers the half-life of erythrocytes, thereby reducing the number of erythrocytes and leading to iron-restricted erythropoiesis. All these factors contribute to the initiation of ACD [8, 10]. Additionally, in ACD patients, impairment occurs in the process of proliferation and differentiation of erythroid progenitor cells such as burst-forming units and erythroid colony-forming units [8]. In the early stage of ACD, the patients have normal iron storage with slight difficulty in iron recycling, and they develop mild, normocytic anemia. However, with time, iron absorption by the intestine is reduced causing shortage of iron notably and subsequent microcytic anemia.

Multiple studies determined the probable role of interleukins and inflammatory cytokines, particularly interleukin-6 (IL-6) in ACD pathogenesis [8, 11]. IL-6 is known to be a multifunctional, pleotropic, and proinflammatory cytokine that is known to be released by immune cells in response to infection or an immunological challenge [3]. The major functions of IL-6 are inflammatory process regulation, immune responses, metabolism of bone, acute-phase reaction, and hematopoiesis [3, 10]. IL-6 inhibits the action of tumor necrosis factor-*α* (TNF-*α*) and thus induces production of ferritin protein [9, 12]. Ferritin is known to be a biochemical marker of iron status in the body, which is upregulated during chronic inflammations [10]. Higher level of ferritin enhances the retention of iron within the reticuloendothelial system. ACD is usually characterized by increased ferritin level [12].

Recently, researchers identified a crucial regulatory agent for iron homeostasis [13] known as hepcidin, which is a 25-amino acid type-II acute-phase protein analogous to ferritin [14]. Inflammation did not result in hypoferremia in hepcidin-deficient mice, indicating the probable role of hepcidin in diversion of iron trafficking resulting in diminished iron absorption in duodenum and prevention of iron discharge from macrophages [8]. Hepcidin is an iron-regulatory peptide hormone with antimicrobial activity generated by human hepatic cells [14] when there is iron overload or inflammation [15], but is reduced in case of iron-restricted erythropoiesis (iron-deficient anemia) [16]. The inflammatory cytokines, such as IL-1*β* and IL-6 induce hepcidin secretion [15]. Hepcidin is associated with iron absorption in the intestine followed by the release of iron from macrophages and hepatocytes [14, 17] via a single biochemical mechanism: hepcidin-ferroportin interaction [18]. Ferroportin is an iron exporter present in the cells. This is used by the reticuloendothelial macrophages and intestinal epithelial cells to export iron from the duodenal enterocytes to the blood [18]. Hepcidin stimulates degradation of ferroportin, thus reduces iron supply to the bone marrow and lowers serum iron levels [19].

Research studies were conducted to develop therapeutic agents inhibiting hepcidin in order to enhance iron utilization from iron storage. Anti-hepcidin antibody was used in mice to validate the efficacy of this agent to inhibit hepcidin [20]. In the first human clinical trial, Van Eijk et al. used an antihepcidin peptide, Spiegelmer lexaptepid, and detected the effectiveness of lexaptepid in preventing systemic inflammations and thus suggested lexaptepid to be a promising therapeutic agent for ACD [21].

The main objective of this study was to assess the role of IL-6 and hepcidin in the development of anemia of chronic disease in Egyptian patients. This will be helpful in the development of novel drugs to inhibit the effect of this inflammatory cytokine and peptide on iron metabolism.

## 2. Materials and Methods

### 2.1. Study Population

This study included 40 subjects diagnosed with ACD (20 with chronic renal failure and 20 hepatic cases). Age of the participants ranged from 60–85 years. There were 14 males and 26 female cases. Forty healthy subjects matched with the age and the sex of the ACD patients were chosen as controls. Both groups were attending the outpatient clinics of Kasr El-Aini Hospital, Faculty of Medicine, Cairo University, Egypt. ACD was described by low hemoglobin (Hb) level and exclusion of other causes of anemia based on clinical and laboratory data. ACD was confirmed by low transferrin saturation, the coexistence of normal or high serum ferritin concentration, and low total iron-binding capacity (TIBC). Patients with other causes of anemia and hypersplenism and those aged below 60 years were excluded.

The study was conducted in accordance with the guidelines approved by the Ethics Committee of Kasr EL-Aini Hospital, Cairo University, Egypt. Written informed consent was also obtained from all the study participants.

### 2.2. Specimen Collection and Evaluation

Whole blood samples were collected in EDTA tubes for determining the hematological markers, such as complete blood count, mean corpuscular hemoglobin (MCH), blood film, Hb, and mean corpuscular hemoglobin concentration (MCHC). Hematological analysis was conducted using Bayer ADVIA 120 (Bayer-Germany) [22]. Serum tubes were separated and frozen till usage for liver and kidney functions, iron profile, and measurement of serum levels of IL-6 and hepcidin. Serum iron was measured by Vitros Chemistry Products Fe Slides (VITROS 5600 Integrated System/2008) (U.S.) [23]. TIBC was measured by Vitros Chemistry Products dTIBC Reagent (VITROS 5600 Integrated System/2008) (U.S.) [24]. IL-6 and hepcidin levels were measured by enzyme-linked immune assay (ELISA) technique [3, 25]. Transferrin saturation was measured by following the equation: [total iron]/[TIBC] × 100 [3].

### 2.3. Statistical Analysis

Student's *t*-test for independent samples was used to compare different study groups. All data were designated in terms of mean ± standard deviation (SD), median and range, or frequencies (number of cases) and percentages when appropriate. A *p* value less than 0.05 was considered to be statistically significant for this study. All the statistical calculations were done using the computer program SPSS (Statistical Package for the Social Science; SPSS Inc., Chicago, IL, USA) release 15 for Microsoft Windows (2006).

## 3. Results

The present study was conducted on 80 subjects: 40 patients and 40 age-matched controls. The characteristics of patients are shown in Table 1. The red cell indices and iron indices for both patients and controls are listed in Table 2. The red cell indices and iron indices show statistically significantly high differences between the cases and the controls. Hb level in cases (8.7 ± 1.5 g/dl) was found to be significantly lower (*p* < 0.001) than that in the control group (13.2 ± 0.9 g/dl). Moreover, some other hematological parameters were observed to be remarkably lower in cases than in controls (*p* < 0.001) including RBC counts (3.3 ± 0.7 in cases and 4.4 ± 0.5 in controls), MCHC (31.4 ± 3.3 vs. 35.65 ± 3.28), HCT (26.7 ± 5 vs. 36.1 ± 3.6), serum iron (62 ± 31 vs. 115.9 ± 31), and serum TIBC (171.2 ± 59 vs. 258.6 ± 41). On the contrary, no statistically significant differences were observed for other blood parameters, such as MCV, MCH, and transferrin saturation % (*p*=0.69, 0.97, and 0.63, respectively).

Screening of hepcidin and IL-6 was performed on all subjects, summarized in Table 3. Highly significant difference was detected between cases and controls. Serum IL-6 (157 ± 2.601 in cases vs. 26.16 ± 27.646) and hepcidin (5.99 ± 2.601 in cases vs. 0.91 ± 1.552 in controls) levels were found to be significantly higher in cases than in controls (*p* < 0.021 and *p* < 0.001, respectively). A nonsignificant correlation (*p* > 0.05) was observed between IL-6 and hepcidin among cases.

## 4. Discussion

Anemia of chronic disease occurs due to reduced erythrocyte production associated with chronic inflammatory conditions [15]. Impaired iron homeostasis and low serum iron level due to increased absorption as well as retention of iron by reticuloendothelial cells, along with low level of erythropoietin and high level of inflammatory cytokines (IL-6) and liver hormone hepcidin, are the characteristic features of ACD [15, 26].

In the present study, the hematological parameters, such as RBC, Hb, and MCHC were observed to have significant differences between ACD patients and healthy controls; however, MCV and MCH showed no significant differences between the two groups. Lower Hb and RBC counts in cases than in controls are due to limited availability of circulating iron and reduced erythropoiesis and lower half-life of RBCs [8, 10]. In fact, according to a past study, ACD development is governed by three immune mechanisms: a reduction in RBC life spans, inability of erythroid cells to proliferate, and increased iron retention in the reticuloendothelial system [10].

In the current study, a high statistically significant difference has been observed between the cases and controls when the immunological indicators were compared. The serum hepcidin and IL-6 levels showed marked increase in cases than in controls. This was in harmony with the earlier study findings [2, 26, 27]. Some previous studies using mouse model or human cell lines reported that IL-6 is a central mediator of chronic inflammation that causes anemia in aged persons [1, 28]. Den Elzen et al. conducted a study on elderly people with anemia of inflammation and detected a higher level of hepcidin in them [2]. The elevation of hepcidin in ACD patients was observed in our study, and other previous studies are thought to be multifactorial involving regulation by iron, inflammation, and demand for erythropoiesis [2, 26]. We also noticed that the mean values of iron indices in cases were considerably less than the controls (*p* < 0.001). The observed finding is analogous to that described in earlier research studies on different populations, which reported strong associations between body iron status, plasma hepcidin levels, and erythropoietin, where higher hepcidin levels were found in subjects with anemia of inflammation [2, 29]. All these data indicated the role of the inflammatory cytokines in stimulating hepcidin expression and thus resulting in low serum iron associated with the inflammatory episodes [27]. During the chronic inflammatory state, immune cells such as macrophages and T-lymphocytes produce more proinflammatory cytokines, especially IL-6, which in turn is responsible for the production of hepcidin [26]. Moreover, a strong association between IL-6 level and disease severity had been reported by a past study [10]. Higher production of IL-6 at inflammatory sites promotes the binding of the signal transducer and activator of transciption 3 (STAT3) transcription factor to the hepcidin gene promoter and upregulates the expression of the hepcidin gene [27]. This induction of hepcidin expression results in hypoferremia, which accompanied inflammatory episodes [26]. In their study, Nemeth and colleagues reported about the necessity of IL-6 in the induction of hepcidin at the time of inflammation and indicated the importance of iron-regulating peptides in the pathogenesis of ACD [28]. Increased hepcidin production stimulated by IL-6 enhances iron retention in reticuloendothelial cells and impairs iron release, thus represses intestinal iron absorption [26, 27] and reduces erythropoiesis leading to anemia. Ferritin production is also increased under the influence of IL-6. Increased ferritin then enhances retention and storage of iron inside the reticuloendothelial cells. In fact, some earlier studies also detected an increase in inflammatory markers, such as erythrocyte sedimentation rate (ESR), ferritin, hsCRP, and leukocytes in ACD patients. This finding supported that IL-6 and the inflammatory markers are positively correlated [10] although we didn't measure inflammatory markers in our study subjects.

Several previous studies had determined a highly significant correlation between hepcidin and IL-6 levels in ACD patients [3, 26, 30]. Contrary to this, the present study detected nonsignificant correlation between IL-6 and hepcidin in the cases, and this could be due to smaller sample size. Moreover, hepcidin level may be increased with age, independent of IL-6, if kidneys fail to clear hepcidin efficiently, or if other inflammatory cytokines, such as IL-1*α* or *β* induce its production, suggesting that IL-6 and hepcidin possibly led to the development of anemia with age independently [1].

Ferruci et al. conducted a study on a large Italian population with ACD and noticed that urinary hepcidin level was not high in subjects with anemia of inflammation compared to nonanemic controls [31]. This was in contrast to our finding. This difference could be due to different underlying diseases with milder inflammatory state or different types of sample used. However, the study by Ferruci et al. confirmed the relationship between high levels of IL-6 and lower serum iron in the cases than in the controls, and this was in accordance with our data.

The present study has few limitations. Firstly, the sample size was not very large and also single centered. Future multi-center studies with large sample size are warranted to infer the existence of a significant link between serum IL-6 and hepcidin levels in ACD patients. Secondly, serum IL-6 and hepcidin were determined at a single time point. Hence, any alteration in their levels, which may appear with time, cannot be considered.

## 5. Conclusion

The current study has shown the importance of considering the levels of IL-6 and hepcidin along with iron profiles in older patients with chronic diseases to distinguish the difference between true iron deficiency and functional iron deficiency. IL-6 is a key mediator of iron-restricted erythropoiesis leading to ACD. Serum hepcidin levels showed correlation with microinflammation and iron status. Hepcidin is considered to have a central role in maintaining overall iron homeostasis in the body and is a long-sought iron-regulatory hormone. Anemia of inflammation is a complex, multifactorial condition, and its exact pathophysiology is not completely understood, thus making its clinical management quite challenging. Recent research studies on IL-6 and hepcidin led to better understanding of ACD pathogenesis. The knowledge on the mechanism of ACD development obtained from the previous research studies, and the findings of the current study may be beneficial in promoting drug development research focusing on hepcidin or IL-6 antagonists. Inhibition of IL-6 or hepcidin probably would improve the utilization of iron from cellular iron storage sites in ACD. This in turn may result in better outcome of ACD management in older patients. Hence, future studies should be directed in identifying more compounds that are able to bind with either hepcidin or IL-6 and prevent their effect on iron metabolism in ACD patients.

## Figures and Tables

**Table 1:** Clinical and laboratory findings of 40 adult anemia of chronic disease (ACD) cases.

Variable	Findings
Age	62.5
Sex	
Male	14
Female	26
Clinical characteristics	
Renal	20 (50)^*∗∗*^
Hepatic	20 (50)^*∗∗*^
Ascites	12 (30)^*∗∗*^
DM	8 (20)^*∗∗*^
HTN	7 (17.5)^*∗∗*^
HCV positive	28 (70)^*∗∗*^
Red cell indices	
Hemoglobin (g/dl)	8.7 ± 1.5
MCV (fl)	80 ± 4.9
MCH (pg)	29.7 ± 2.9
MCHC (g/dl)	31.3 ± 3.2
Red blood cells (×10^6^/*μ*l)	3.27 ± 0.76
HCT (%)	26.7 ± 5.1
Iron indices	
Serum iron (*μ*g/ml)	62.28 ± 31.4
TIBC (*μ*g/ml)	171.18 ± 59.27
Transferrin saturation%	43.3 ± 32.76
Serum hepcidin and IL-6	
Hepcidin (ng/ml)	5.9 ± 2.6
IL-6 (pg/ml)	157 ± 35

^*∗*^RBCs: red blood cells; MCH: mean corpuscular hemoglobin; MCV: mean corpuscular volume; MCHC: mean corpuscular hemoglobin concentration; HCT: hematocrit; TIBC: Total iron-binding capacity. ^*∗∗*^Number (%).

**Table 2:** Comparison statistics of the red cell indices and iron indices in both cases and controls.

Variable	Controls (*n* = 40)	Cases (*n* = 40)	*p* value
	Mean ± SD	Mean ± SD	
Hemoglobin (g/dl)	13.2 ± 0.9	8.7 ± 1.5	0
MCV^*∗*^ (fl)	82.6 ± 3.9	80 ± 14.9	0.696
MCH^*∗*^ (pg)	29.7 ± 2.6	29.7 ± 3.9	0.979
MCHC^*∗*^ (g/dl)	35.65 ± 3.28	31.4 ± 3.3	0
RBCs^*∗*^ (×10^6^/*μ*l)	4.4 ± 0.5	3.3 ± 0.7	0
HCT^*∗*^ (%)	36.1 ± 3.6	26.7 ± 5	0
Serum iron (*μ*g/ml)	115.9 ± 31	62 ± 31	0
Serum TIBC (*μ*g/ml)	258.6 ± 41	171.2 ± 59	0
Transferrin saturation %	40.7 ± 9.7	43.3 ± 33	0.634

^*∗*^ *p* value <0.05 is taken as significant.

**Table 3:** Comparison statistics of the serum hepcidin and IL-6 in both cases and controls.

Variable	Controls (*n* = 40)	Cases (*n* = 40)	*p* value
	Mean ± SD	Mean ± SD	
Hepcidin (ng/ml)	0.91 ± 1.552	5.99 ± 2.601	0.000
IL-6 (pg/ml)	26.16 ± 27.646	157 ± 349.244	0.021

^*∗*^ *p* value is significant if < 0.05.

## Data Availability

The data used to support the findings of this study are included within the article.
